# NDVI and vegetation volume as predictors of urban bird diversity

**DOI:** 10.1038/s41598-025-96098-0

**Published:** 2025-04-15

**Authors:** Andrew J. Fairbairn, Sophia Katholnigg, Tobias Leichtle, Lisa Merkens, Louis Schroll, Wolfgang W. Weisser, Sebastian T. Meyer

**Affiliations:** 1 Terrestrial Ecology Research Group, Department of Life Science Systems, School of Life Sciences, Technical University of Munich, 85354 Freising, Germany; 2 German Remote Sensing Data Center, German Aerospace Center, 82234 Oberpfaffenhofen, Germany; 3 Studio Animal-Aided Design, 10999 Berlin, Germany; 4 École Normale Supérieure de Lyon, Université Claude Bernard Lyon 1, Université de Lyon, 69342 Lyon Cedex 07, France

**Keywords:** NDVI, Urban ecology, Remote sensing, Biodiversity, Urban ecology, Urban ecology, Biodiversity

## Abstract

Urban expansion and densification pose a challenge to urban biodiversity. Rapid estimation of biodiversity could help urban planners balance development and conservation goals. While the Normalised Difference Vegetation Index (NDVI) has proven useful for predicting urban bird diversity, new products derived from remote sensing, such as vegetation volume, could provide more detailed descriptions of available habitat, potentially improving biodiversity predictions. We evaluated the effectiveness of NDVI and vegetation volume as predictors of urban bird diversity and local community composition for different buffers around 86 sampling points in Munich, Germany. Using linear models, we showed that a 100 m buffer best described bird diversity (highest R^2^) for both NDVI and vegetation volume compared to the other buffers. Contrary to expectations, NDVI was better than vegetation volume in predicting bird diversity (mean R^2^ NDVI = 0.47, mean R^2^ vegetation volume 0.37). We found a shift in community composition from species associated with human-modified landscapes to those associated with forests along an urban greenness gradient. In contrast to diversity, we found that vegetation volume was slightly better at predicting community composition. Using NDVI to predict bird diversity across Munich, we demonstrated its potential for predicting city-wide bird diversity. We discuss how such predictive maps can be used for urban planning and conservation. As urbanisation continues to impact global biodiversity, refining ecological models for urban planning will be crucial to developing more biodiverse urban environments.

**Supplementary Information:**

The online version contains supplementary material available at 10.1038/s41598-025-96098-0.

## Introduction

Currently, over half of the global population resides in urban areas, with projections indicating that this figure will reach nearly 60% or more before the end of the century^1^. This rapid urbanisation is reducing the amount of nature and green accessible to urban residents. There is a growing awareness of the importance of urban nature, both for human well-being^2–5^ and its role in maintaining biodiversity^6^. Indeed, as much as 50% of the regional biodiversity can also be found in cities^7^, and cities can serve as habitats for rare or endangered species^8,9^. Given that urban nature is often the only nature that city residents are exposed to on a daily basis, it plays a crucial role in connecting people with nature while simultaneously supporting diverse species and contributing to local and global conservation efforts. Birds, in particular, contribute significantly to urban residents’ daily nature experiences and are indicators of overall biodiversity^10^ but also.

To preserve and enhance urban biodiversity, urban planners and conservationists can prioritise biodiversity in both the planning process and ongoing conservation efforts^11,12^. This can be achieved through various strategies, biodiversity-rich urban sites can be strategically protected from densification^13^, and stepping stones can be established to make more resources accessible to support urban biodiversity^14^. Conservation strategies targeting urban birds can include creating and maintaining diverse green spaces, preserving mature trees, installing artificial nest boxes, and reducing threats such as window collisions and outdoor cats^15,16^. However, for biodiversity conservation to become integrated into urban planning, more technical knowledge about where biodiversity is high and how to estimate it is needed^17^. Therefore, providing planners and conservation practitioners with a simple tool to estimate the biodiversity of urban sites and assess the potential impact of various interventions could help them predict the outcomes of planning and conservation actions and prioritise urban biodiversity conservation. This need for new, simple and rapid biodiversity assessment tools is particularly evident when considering the drawbacks of current monitoring methods.

Traditional approaches to monitoring urban birds typically use field methods such as point counts or transects with recent years seeing increased adoption of acoustic monitoring. Advancements in bioacoustics, computing, and machine learning, particularly through tools such as BirdNET^18^, now allow for the simultaneous monitoring of hundreds of sites. In Germany, BirdNET has coverage for 407 out of 527 species tracked by the German Ornithologists Society^19^, including rare or incidental species and can detect species confidently up to and beyond 50 m from recording devices^20^. BirdNET has proven itself as the de facto standard, being successfully tested and employed in a variety of environments to a high degree of accuracy^21–26^.

However, with bioacoustics, unlike point counts, measures of abundance are not readily available. To address this limitation, ecologists have examined acoustic-based metrics as proxies for abundance particularly Vocal Activity Rate (VAR). VAR and variations of it have been demonstrated to be a good proxy for abundance for a number of species^27–31^. However, the context is also important for the goodness of fit; for example, VAR has been shown to be well suited for estimating seabird population size^27,30^ and territorial songbird abundance^29^, but for solitary raptors, it may reflect behavioural changes rather than abundance^31^. In addition, a short recording length, the species’ behaviour, the time of year, and the surrounding environment can influence the reliability of these measurements. For example, short-term changes in vocal activity may influence VAR when derived from short recordings^29,31^.

Traditional and acoustic monitoring methods can provide an accurate and detailed picture of urban bird diversity at single sites. However, a city-wide scale monitoring of diversity remains challenging because measurements are time-consuming and require considerable expertise in bird identification. These limitations have prompted researchers to explore alternative approaches for rapidly assessing urban diversity using indirect proxies based on a small number of monitoring sites. Models based on multispectral satellite imagery have emerged as a promising tool in this regard. Specifically, vegetation indices derived from satellite data, such as the Normalised Difference Vegetation Index (NDVI), have been shown to be predictive for various urban bird diversity metrics^32–35^.

NDVI quantifies the ratio of the red and infrared reflectance of diverse surfaces, which is correlated with certain physical properties of the vegetation canopy: leaf area index (LAI), fractional vegetation cover, vegetation condition, and biomass^36^. While NDVI can be employed to describe and classify diverse vegetation types when utilising time series (e.g^37–39^). , a single snapshot or average across time of NDVI may only indicate the presence of vegetation in contrast to other surfaces with different reflectance values. While NDVI can provide valuable information about the physiological status of vegetation, such as stress, phenology, and vegetation density^40^, it provides limited insights into other attributes of urban vegetation, such as height or type. Although NDVI has been established as an effective measure of urban green^41^, which is generally correlated with higher biodiversity, the specific components that comprise this green and the characteristics of the remainder of the urban matrix significantly influence the diversity of an area^42^. Thus, for predicting the diversity of birds, exploring additional data derived from remote sensing is crucial for comprehensively understanding urban biodiversity and providing practitioners with accurate tools for rapidly estimating biodiversity.

While other common metrics, such as Leaf Area Index (LAI) or Enhanced Vegetation Index (EVI), may be considered by some, LAI is not readily available globally at very high resolutions, and EVI has been found by others to not be as good as NDVI for predicting different aspects of bird diversity in cities^32^. A three-dimensional approach to vegetation assessment could be particularly relevant to bird diversity studies, as different species utilise multiple and varied vegetation strata^43^, and suitable vegetation volume and density are important for maintaining native bird communities^44^. Recent advances in satellite and aerial imagery technologies, coupled with improvements in digital surface models, have opened new possibilities for describing urban vegetation in more dimensions. One particularly promising metric is vegetation volume, which includes both the area and height of vegetation. Further, bird species respond differently to vegetation composition and structure, with varying responses between species and functional groups^45,46^. Incorporating height adds another dimension that should better describe the habitat available to birds. For instance, areas with similar NDVI values might have vastly different vertical structures and, therefore, vegetation volume, which could influence local bird diversity. Consequently, vegetation volume likely offers a more accurate prediction of urban bird diversity than NDVI.

We aim to evaluate the efficacy of NDVI and vegetation volume for describing bird biodiversity in Munich, Germany and discuss the utility of vegetation index proxies of bird diversity for urban planning. Specifically, we examine the extent to which we can describe bird richness, vocal activity, diversity and community composition using NDVI and vegetation volume. Our objectives are to (a) determine the optimal buffer around each point that best describes the species recorded there, (b) compare NDVI and vegetation volume as proxies for bird diversity, and (c) examine how well NDVI and vegetation volume represent local community composition. Using these findings, we generate predictions of bird diversity across Munich and discuss applications for urban planning and biodiversity conservation.

## Methods

### Site selection

**Fig. 1:**
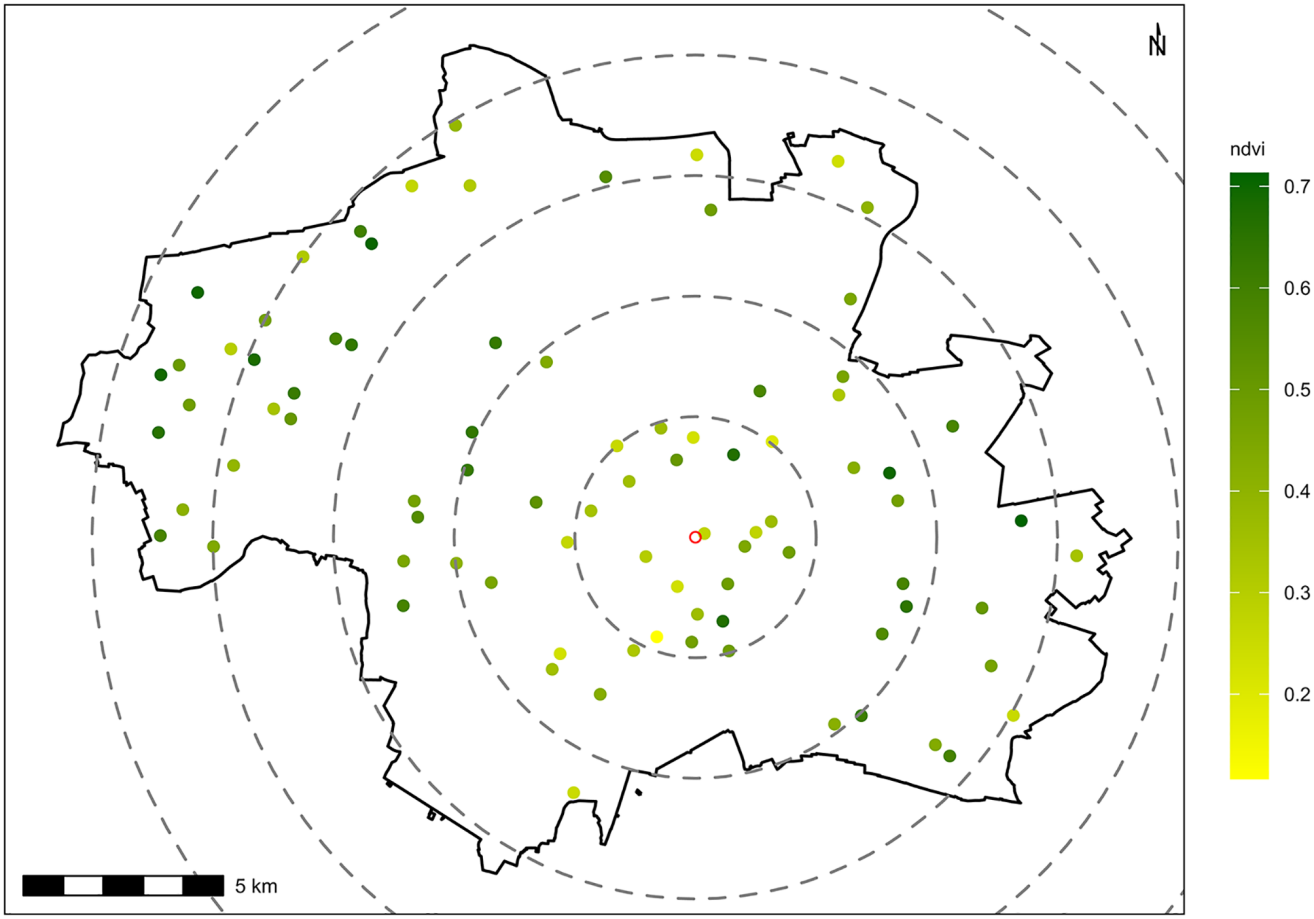
Spatial distribution and NDVI of acoustic monitoring points in Munich, Germany. Map of 86 points in the City of Munich, Bavaria, Germany, where acoustic monitoring of birds was undertaken. The green gradient represents the average NDVI value within a 100 m buffer around each point. The open red dot is the historic city centre, Marienplatz. Grey dashed circles represent three-kilometre-wide rings within which the point selection was undertaken.

We selected 86 sites in Munich, Bavaria, southern Germany, spanning two orthogonal gradients in NDVI and distance from the city centre (Marienplatz, latitude 48.137, longitude 11.576). Starting with a very high resolution 20 × 20 cm pixel NDVI map of Munich derived from data obtained from the Bavarian State Office for Digitization, Broadband and Surveying (https://www.ldbv.bayern.de/index.html), we aggregated the map to 100 m (*aggregate* function *terra* package in R^47^) and zeroed all negative values to reduce the range of non-vegetated pixels. We created five even bins spanning the range of NDVI values (0 to 0.71). We then separated the city into five three-kilometre distance rings (0–3 km, 3–6 km, 6–9 km, 9–12 km, 12–15 km) around the city centre. Next, we conducted a random stratified sampling to select 100 raster cells, with ten sites per distance ring and two per NDVI, with the constraint that there was a minimum of 300 m between sites to ensure independence^48^ (Fig. 1). Raster cells where more than 25% of the cell were outside of the city boundary were removed, resulting in 86 cells. We selected a streetlamp closest to the centre of each selected raster cell as the sampling point using Google Maps and on-site surveys.

### Bird surveys

We deployed a single Frontier Labs Bioacoustic Recorder (BAR) or BAR long-term (BAR_LT) on each sampling point. Recorders were set to record for one week, 24 h a day, with a sample rate of 48 kHz and a gain of 40. Recorders were deployed at a standardised height of approximately 4 m. We used 25 recorders and needed four weeks for recording (recording started on weeks 14, 27, 29, and 31 of 2023). Each sampling point was sampled once. A continuous and long recording period increases the likelihood of detecting species that may vocalise infrequently or would be missed by shorter schedules or short, infrequent visits, as with point counts^26^. However, like any ecological survey method, is subject to biases. Specifically, the detectability of species can vary due to multiple factors, including the structure of the surrounding environment, ambient noise, weather conditions, and distance from the microphone.

### Bird diversity metrics

To calculate bird richness, activity, and diversity, we analysed all the recordings with BirdNET-Analyzer^18^ (v2.4), including the week of the year, location, a default sensitivity of 1.0, a default overlap of 0 s and the results filtered by a minimum confidence of 0.8 following Fairbairn et al.^26^. We removed singletons (species only detected on an individual site once) as they are likely incidental and the species not a resident of that site. All species detected ten times or fewer overall were manually checked, and incorrect identifications were removed. The filtered and validated results were compared to species occurrence data since 2000 from the Global Biodiversity Information Facility (GBIF) using the *rgbif* package in R^49,50^ and the NABU (Naturschutzbund Deutschland e.V.) 40 most common garden birds (https://www.nabu.de/tiere-und-pflanzen/aktionen-und-projekte/stunde-der-gartenvoegel/portraets/index.html). We then calculated the species richness per site by summing all species detected. As a measure of vocal activity, which can be used to approximate relative abundance, we calculated the vocal activity rate (VAR, Eq. 1) as the ratio of the number of 15-minute periods a species was detected vocalising at least once to the total number of days recorded.

1 $$\:VAR=\frac{n\:15-minute\:periods}{days\:recorded}$$

While VAR is typically calculated as the total number of vocalisations of a species over the recording length, we count multiple vocalisations only once in a 15-minute period in an effort to reduce the overestimation of species that vocalise frequently^31^. We summed the by-species vocal activity rates to get a VAR per site. It should be noted that while our implementation of VAR may be more robust^31^, acoustic abundance indices can be affected by the recording length and schedule and individual species’ life histories^29,31^ as such it may not be a direct proxy for abundance for some species. Finally, we calculated the Shannon diversity index for each site by using the VAR of a species in place of abundance.

### Mapping NDVI and vegetation volume

**Fig. 2:**
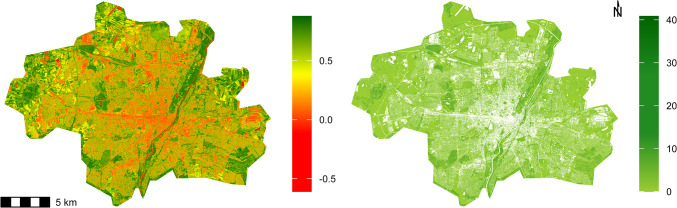
NDVI and vegetation volume maps of Munich, Germany. (**a**) 30 cm resolution NDVI map (**b**) 50 cm resolution green volume map. Maps plotted at 5 m resolution.

While site selection was done on an NDVI map from 2017, we obtained newer remote sensing data from 2019 for all further analyses. Optical satellite imagery with very high spatial resolution of 30 × 30 cm^2^ was acquired by WorldView-3 on 04.07.2019. Based on these data, the NDVI was calculated (range − 1 to 1, Fig. 2) for the single date. We also calculated the Enhanced Vegetation Index (EVI) as an additional vegetation index based on the WorldView-3 data^51^. However, EVI was strongly correlated with NDVI (Pearson’s *r* = 0.99; Supplementary Fig. S1), so we retained NDVI as the primary vegetation index for further analyses.

Using the WorldView-3 imagery, a normalised digital surface model (nDSM) with very high spatial resolution of 50 × 50 cm² and data from OpenStreetMap, a land cover map was generated based on the approach presented in Taubenböck et al.^52^ and Wurm et al.^53^. For the calculation of vegetation volume, all vegetated pixels were extracted from the land cover map, and vegetation volume in m³ was calculated based on the volume below the surface model (i.e. representation of the vegetation canopy) provided through the nDSM (Range 0 to 41, Fig. 2.).

### Site characteristics at differing Spatial scales

To characterise the area surrounding each sampling point, we calculated mean NDVI and mean vegetation volume (henceforth NDVI and vegetation volume) within several buffers around each sampling point (25 m, 50 m, 100 m, 200 m, 400 m, 800 m). These buffer sizes were chosen to capture local variation in vegetation characteristics at scales relevant to urban planning (e.g. within a city block^54^ or urban square^42^) and typical home ranges of urban bird species^55,56^ while also including the distances typically used in traditional point counts. Mean NDVI was calculated using the *extract* function in the *terra* package^47^ using a summary function of mean. Mean vegetation volume was calculated similarly, using a sum function and dividing the total vegetation volume by the buffer area for each buffer to account for NA values in the non-vegetated pixels (Supplementary Table S1). To meet the linearity assumptions of the linear models, vegetation volume was then log-transformed for all analyses.

**Fig. 3:**
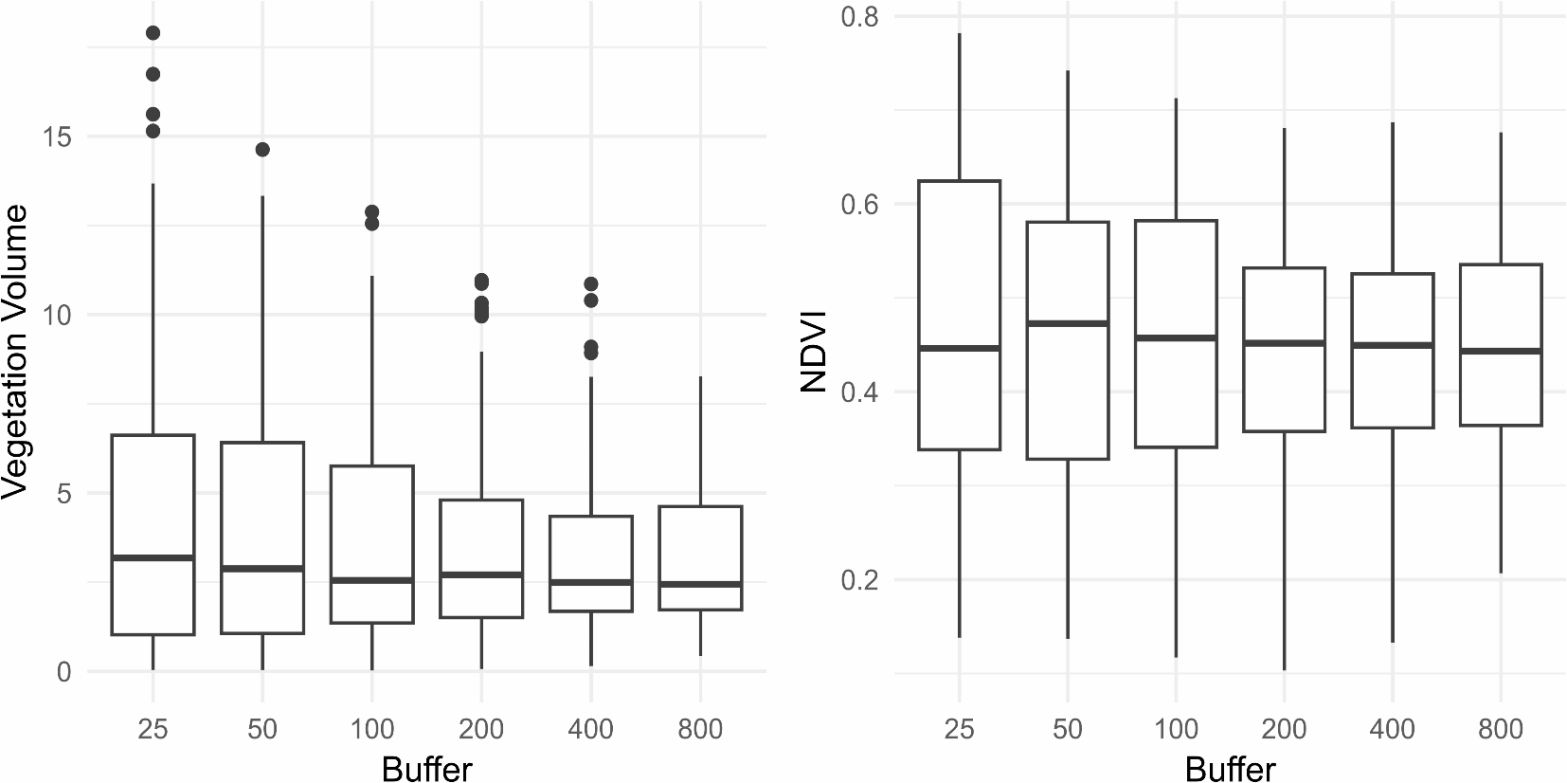
Variation in NDVI and vegetation volume around monitoring points. Boxplots showing the distribution of mean NDVI (left) and mean vegetation volume (right) at six different radius buffers (25, 50, 100, 200, 400, 800 m) around 86 bird monitoring points in Munich, Germany.

Minimum and maximum values for NDVI and vegetation volume varied considerably across buffers (Fig. 3). NDVI ranged from 0.103 to 0.782 across all buffers, with the lowest minimum observed at the 200 m buffer and the highest maximum at the 25 m buffer. For vegetation volume, the range was more pronounced, spanning from 0.026 to 17.898 m³/m², with the lowest minimum at the 100 m buffer and the highest maximum at the 25 m buffer (Fig. 3). Both NDVI and vegetation volume generally showed lower range and mean values as the size buffer increased; however, the median value remained similar. NDVI and log vegetation volume correlate at small buffers, but this decreased with increasing buffer size (Supplementary Fig. S2).

### Analysis

To determine what buffer around each point best describes the bird diversity metrics (richness, VAR, and diversity), we compared the partial r-squared (*partial*_r2 from the *sensemakr* package^57^) from linear models for each diversity metric and buffer (bi) for both NDVI and vegetation volume as explanatory variables and recording week and distance from the city centre as covariates to account for temporal and spatial variation (Eq. 2). To ensure the independence of predictors and covariates, we checked for collinearity using the *vifstep* function from the *usdm* package^58^, ensuring a variance inflation factor of less than 5 was maintained. All models were assessed to ensure they met the assumptions of linear regression, including linearity, homoscedasticity, normality of residuals, and absence of multicollinearity. Vegetation volume was log-transformed for all models and NDVI was transformed for the Shannon diversity models. We selected the best buffer as the one with the highest r-squared, which appeared most frequently across all diversity metrics.

2 $$\:lm(diversity\:metric\:\sim\:week+distance\:to\:city\:centre+{\left(ndvi\:or\:vegetaiton\:volume\right)}_{bi}$$

To compare NDVI and vegetation volume as predictors for bird diversity, we compared the best buffer models from before for each diversity metric and NDVI and vegetation volume based on the model r-squared values. The models with the highest r-squared were selected. We tested for spatial autocorrelation in the best models using Moran’s I test implemented in the DHARMa package^59^ to ensure the spatial independence of our observations.

To test how much of the total variation in community composition (the diversity and abundance of different species within a site) can be explained by NDVI and vegetation volume, we utilised non-metric multidimensional scaling (NMDS; *metaMDS* function in the *vegan* package^60^) using VAR as a proxy for abundance and a Bray-Curtis dissimilarity matrix with a log plus one transformation to reduce the effect of highly abundant (vocal) species. To compare if NDVI or vegetation volume best describes community composition, we conducted an environmental fit (*envfit* function of the *vegan* package), including NDVI, vegetation volume, recording week, and distance to the city centre as environmental variables. The NMDS was rotated to align NDVI with NMDS1 for visualisation purposes. We compared variables based on their r-squared and significance. To understand how species habitat preference drives community composition, we assigned each identified species a habitat preference using the AVONET database^61^. Species with a habitat preference for rock were reassigned to human-modified as many urban species would originate from cliff and mountain habitats^62^. Additionally, as Munich is inland, any species with a coastal habitat preference were also reassigned to human-modified. Species associated with water (riverine & wetland) were combined into a single category aquatic, and open land species (grassland & shrubland) were combined into a single category open. The species scores were then plotted in the ordination plot, and their names were coloured by habitat preference for visual inspection of patterns.

As VAR can be hard to compare between bird species because they depend not only on the abundance of species but also on how frequently they vocalize^31^, we assessed how much the patterns observed in the NMDS were driven by vocal activity rate (VAR), we conducted an additional NMDS with the community matrix converted to presence-absence using the *decostand* function from the vegan package and Jaccard distance. To further compare the spatial relationships between the two ordinations, we performed a Procrustes analysis and applied the Mantel test (*procrustes* and *mantel* functions in *vegan*) to assess the alignment and correlation between the Bray-Curtis distance matrix (from the VAR-based NMDS) and the Jaccard distance matrix (from the presence-absence NMDS).

Finally, we predicted all three diversity metrics across Munich using the spatial data we determined as the best predictor. We first split the data using an 80/20 train (*n* = 69)/test (*n* = 17) split. Using the training data, we produced linear models for each diversity metric, including covariates (Eq. 2). We then mapped the results by aggregating the NDVI map to a 100 m resolution (*aggregate* function *terra* package in R^47^) and calculated the distance from the city centre for each cell. Finally, we predicted the three diversity metrics averaging over the temporal block (week of recording) for each 100 m grid cell.

To evaluate the predictive models, we used the test data as new data in the predict function from the *stats* package in base R. To quantify the average discrepancies between the actual and predicted values, we calculated the R Mean Squared Error (RMSE) and the Mean Absolute Error (MAE) for each model. As an additional test of model fit, we conducted a Standardized (reduced) major axis model II linear regression (*lmodel2* function *lmodel2* package in R^63^) for each set of predicted and observed values in the test dataset.

## Results

### Recording and bird results

After cleaning and validating 13,399 h of recordings from 86 sites (73 sites with at least 6 full days of recording, 66 with the full seven; mean 6.49 ± 1.11 days per site), we obtained a total of about 337,000 detections of 86 bird species across all sites (Supplementary Table S2). Species richness on our observation points ranged from 5 to 48 (mean 27.06 ± 9.95). Vocal activity rate ranged from 2 to 273 (mean 91.99 ± 66.01), while Shannon diversity ranged from 1.06 to 3.05 (mean 2.46 ± 0.47)(Supplementary Table S1). Of the 86 species we detected, 85 are also found in the GBIF data, representing a high overlap between the datasets. However, GBIF lists 106 additional species that were not detected in our study. Notably, nearly 50% of these are aquatic species, a habitat type not targeted by our monitoring. Additionally, our results contain 39 of the 40 most common garden birds listed by NABU. The species observed on most sites were the Carrion crow *Corvus corone* (*n* = 78 sites) and the Green woodpecker *Picis viridis* (*n* = 78 sites). The species with the highest vocal activity rates the Yellowhammer *Emberiza citrinella* (mean VAR = 13.43), followed by the Common chiffchaff (mean VAR = 8.78; Supplementary Table S2). The mean VAR per site provides an estimate of how frequently a species is vocalizing, on average, at each site where it is present. Given that there are 96 15-minute periods in a full day, this means that, on average, the Yellowhammer vocalized during about 13 of those intervals at each site where it was detected.

### Optimal buffer for vegetation metrics

**Fig. 4:**
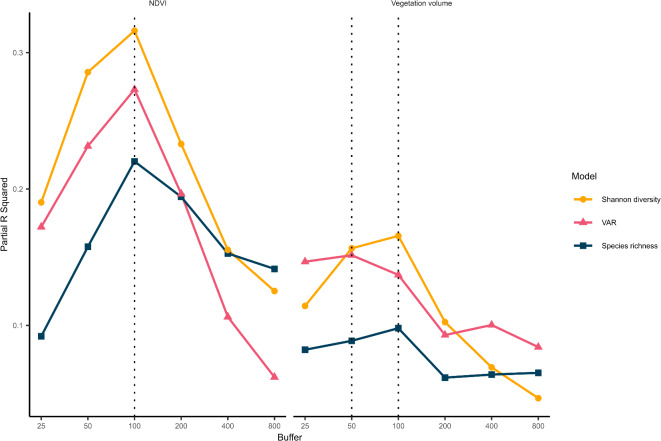
Buffer size optimization using partial r-squared for diversity metrics. R-squared-based best buffer selection for models based on mean NDVI and log mean vegetation volume at six buffers around each observation point for each of three diversity metrics. The y-axis is the partial r-squared for log vegetation volume and NDVI. Dashed lines represent the best-performing buffer based on r-squared. NDVI is log-transformed in the Shannon diversity models.

How well NDVI and vegetation volume could predict properties of the bird community in Munich depended on the buffer around sampling points over which the measures of vegetation were considered. This was indicated by substantial differences in r-squared values across linear models with varying buffers. Generally, diversity metrics were best predicted by vegetation variables at intermediate buffers. For all diversity metrics, we identified a 100 m buffer around each point as the optimal buffer (Fig. 4). Although the 50 m buffer initially appeared preferable for VAR and vegetation volume, we ultimately selected the 100 m buffer for consistency. We decided this for two reasons: the difference in model performance between 50 m and 100 m buffer for vegetation volume was minimal, and maintaining consistency across variables facilitated easier comparison of results (Fig. 4., Supplementary Table S3).

### NDVI and vegetation volume as predictors of bird diversity

**Fig. 5:**
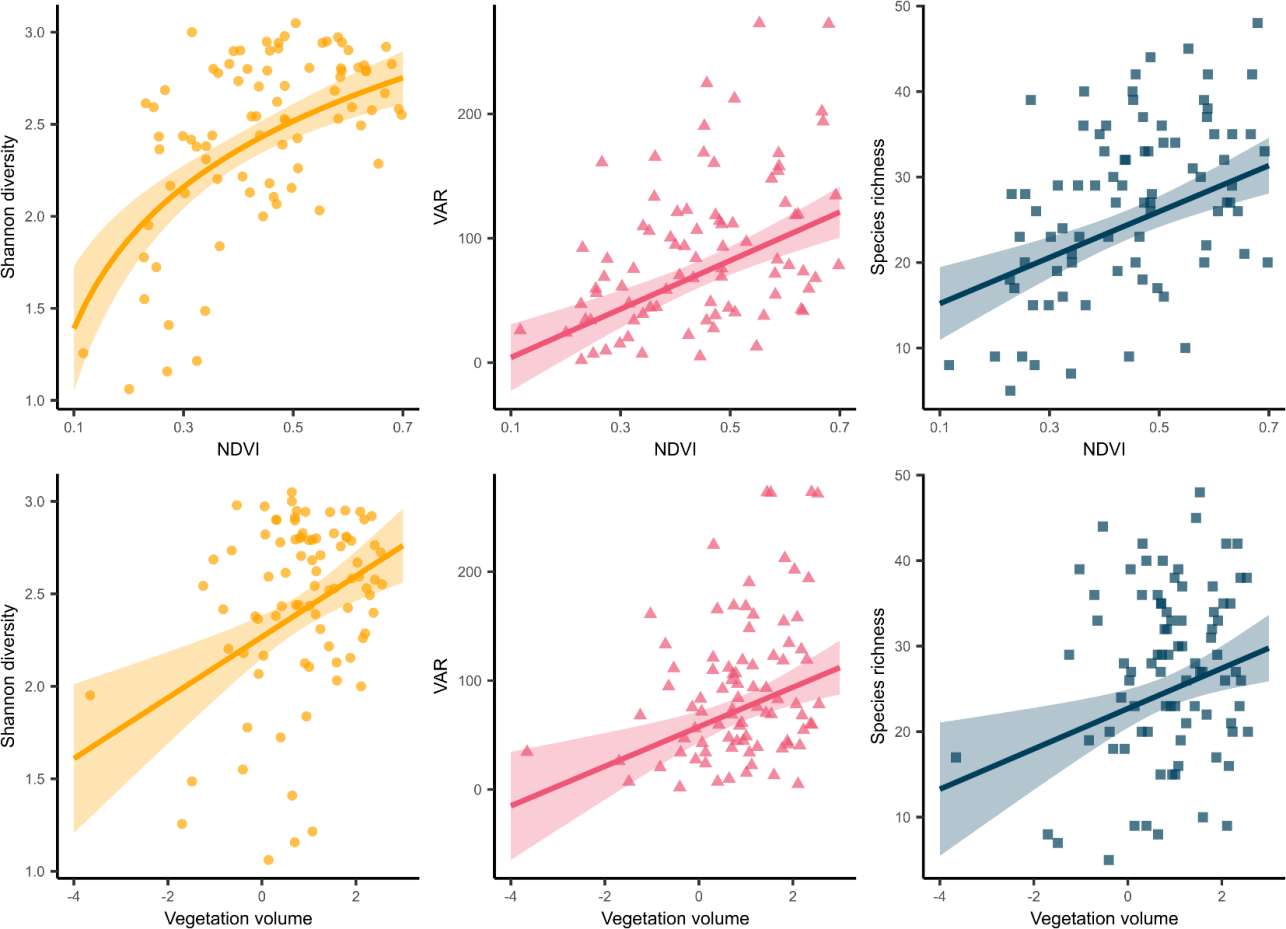
The performance of NDVI and vegetation volume as predictors for bird diversity in Munich, Germany. Each point represents one of 86 points surveyed between March and August 2023. All models are significant (*p* < = 0.05). Lines are model predictions; shaded areas are 95% confidence intervals. Note the logged axis for vegetation volume. NDVI is log-transformed for the Shannon diversity model.

For the 100 m buffer, the best-performing models for all biodiversity metrics were those based on mean NDVI, explaining 23.6% more of the variance in the data than vegetation volume (mean R^2^ NDVI = 0.47 mean R^2^ vegetation volume 0.37; Fig. 4, Supplementary Table S3). Partial R^2^ were also higher for NDVI models (partial R^2^ = 0.22–0.32) than vegetation volume models (0.10–0.17)(Fig. 4). No spatial autocorrelation was found for any of the best models (DHARMa Moran’s I test, *p* ≥ 0.14 for all models). All measures of bird diversity increased with higher values of NDVI and vegetation volume, with species richness and Shannon diversity about doubling and VAR increasing from approximately zero to 100 over the range of the two greenness measures (Fig. 5), which performed best predicting VAR (R^2^ = 0.53, 0.45) followed by species richness (R^2^ = 0.48, 0.40) and then Shannon diversity (R^2^ = 0.40, 0.27).

### Community composition

**Fig. 6:**
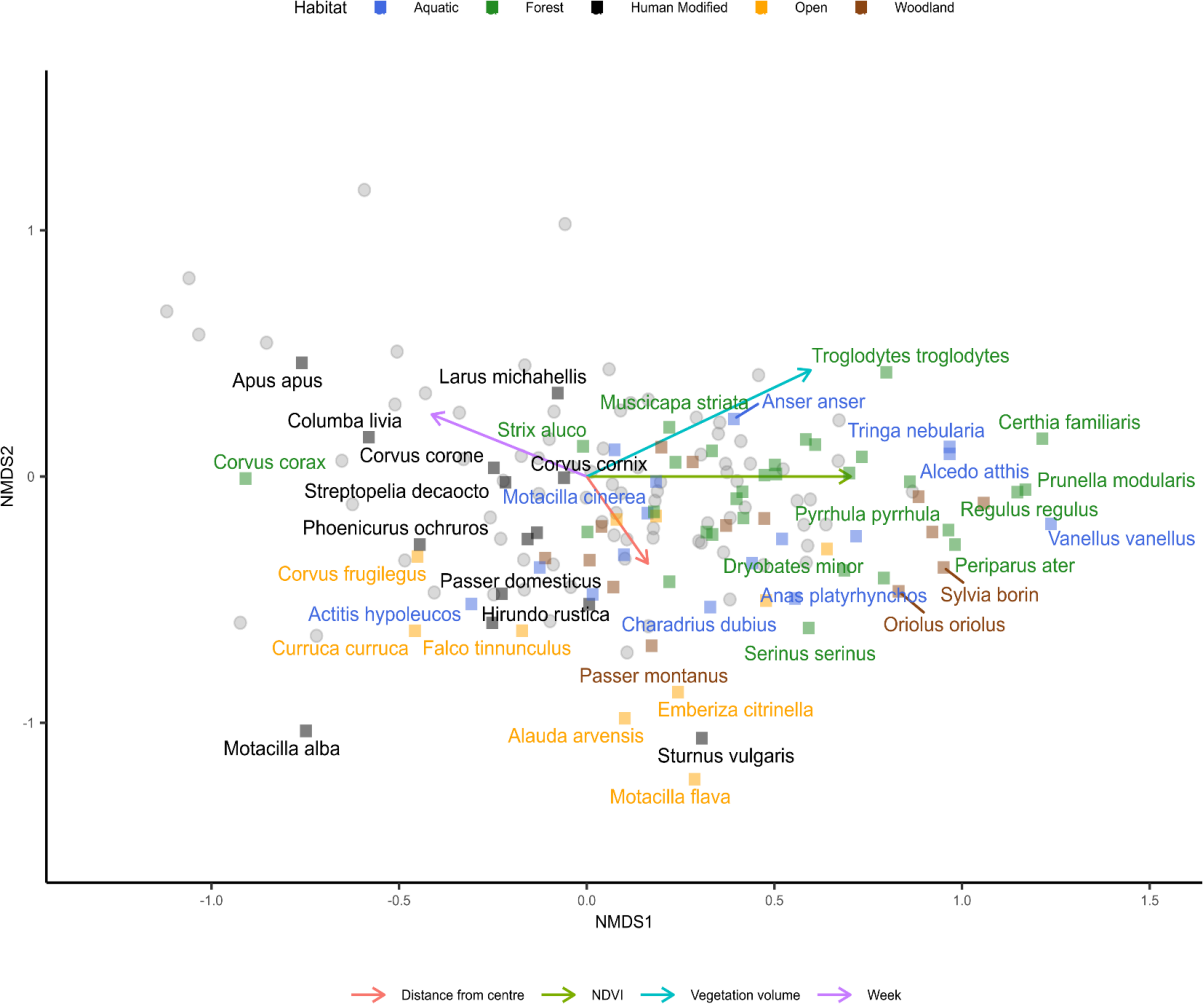
Community dissimilarity across NDVI and vegetation volume gradients in Munich, Germany. Non-metric multidimensional scaling (NMDS) ordination plot with environmental variables indicating the dissimilarity of communities across green mean NDVI and blue log total vegetation volume for 86 sites in Munich, Germany. Grey points represent sites. Species points and labels are coloured by their dominant habitat type. Not all sites or species names are displayed.

Our ordination (R^2^ = 0.61, stress = 0.19) showed a clear shift in community composition among the sampling sites. Species that occurred mostly at high values of nmds1 typically inhabit forest habitats (Fig. 6, dark green), such as the Eurasian treecreeper, *Certhia familiaris*, the Eurasian bullfinch, *Pyrrhula pyrrhula* and the Dunnock *Prunella modularis*. These species cluster to the right of the ordination. In contrast, species on the other end of NMDS1 are more associated with human-modified landscapes (Fig. 6, black), such as agricultural lands and urban areas, including the Western jackdaw *Corvus monedula*, the Barn swallow *Hyrundo rustica*, and the Eurasian magpie *Pica pica*. The central area of the ordination space is occupied by those with intermediate habitat preferences (open woodland, parks, etc.), suggesting a continuous gradient of vegetation and particularly tree dependence across the bird community (Fig. 6). To ensure that these patterns were not driven solely by differences in vocal activity among species, we conducted an additional NMDS using a presence-absence transformation of the community matrix (Supplementary Fig. S2). The resulting ordination (R^2^ = 0.50, stress = 0.20) showed a similar structure, with species composition and habitat-preference shifting along NMDS1 in response to vegetation gradients. Further validation of these patterns through Procrustes analysis and the Mantel test revealed a strong correlation between the ordinations and the distance matrices (Supplementary Information S1).

The environmental fit supports this pattern, showing significant relationships between NDVI, vegetation volume, and community composition (Fig. 6). Due to the rotation, the NMDS1 axis primarily corresponded to a gradient of increasing vegetation volume and NDVI, reflecting a transition from open and human-modified habitats to more densely vegetated habitats with trees. NMDS2 may reflect distance from the city centre, shifting community over time, and other unmeasured environmental factors. The vectors for NDVI and vegetation volume from the environmental fit pointed strongly towards the upper right quadrant of the ordination space, aligning with a gradient in habitat preferences of the bird species. Unlike for the measures of bird diversity, vegetation volume showed a slightly stronger association with community composition (*r* = 0.54, *p* < 0.001) compared to NDVI (*r* = 0.50, *p* < 0.001). While vegetation emerged as the primary driver of community composition, other factors, such as distance from the city centre (*r* = 0.15, *p* < 0.001), also showed a significant, albeit weaker, relationship.

### Predictive modelling results

**Fig. 7:**
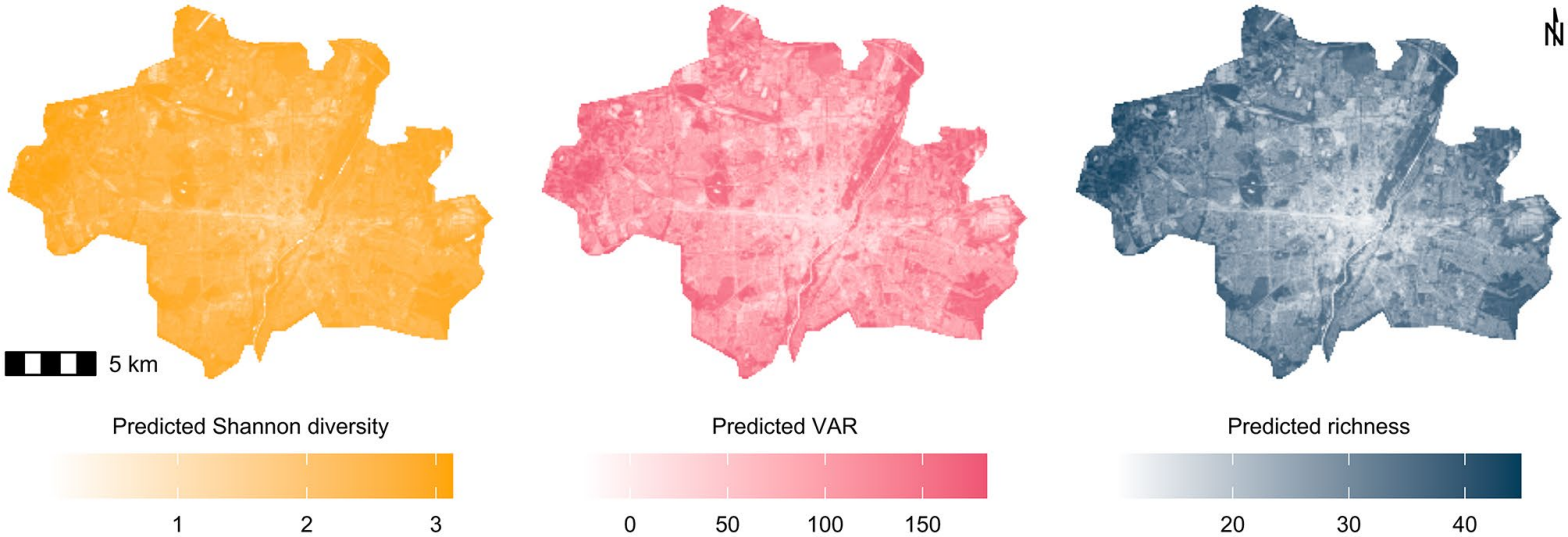
Maps for three bird diversity metrics (richness, VAR, and Shannon diversity) in Munich, Germany. Predictions from linear models for the relationship between bird diversity and NDVI based on 69 measured points.

Our predictive maps for all diversity metrics showed clear distinctions between the heavily populated or developed areas, for example, near the city centre (white) and the greener regions (coloured). Species richness, VAR, and Shannon diversity tended to increase further from the city centre with notable variability and some exceptions of high bird diversity also relatively close to the city centre, such as the English Garden northeast of the city centre (Fig. 7). Given the inherent complexity and variability in ecological systems the models fit the training data well (r^2^ 0.37–0.49, Supplementary Table S4). The evaluation of the predictive models showed good correlation with the actual values; however, there was some variability in the predictions. The root mean square error (RMSE) and mean absolute error (MAE) were as follows: RMSE (richness = 7.82, VAR = 48.22, Shannon = 0.36) and MAE (richness = 6.45, VAR = 40.52, Shannon = 0.30). The standardised (reduced) major axis model II linear regression lines were plotted against a 45-degree line representing perfect correlation (Supplementary Fig. S3). The observed deviations from this line indicated discrepancies between the actual and predicted values. All models showed a significant relationship between the predicted and actual values (Supplementary Table S5).

## Discussion

Our study represents a test case for a spatial product other than NDVI as a predictor for urban bird diversity. We determined that a 100 m buffer around the observation points best described the bird diversity occurring there when bird communities were characterised using passive acoustic monitoring. Contrary to what we expected, we found vegetation volume was not a better predictor than NDVI, which performed better for every diversity metric calculated. However, vegetation volume performed slightly better in predicting community composition than NDVI. These results lend further support to other studies^32,34^, which showed NDVI to be a good predictor for urban bird diversity.

### Optimal buffer for vegetation metrics

We found that a 100 m buffer around our observation points best described the bird diversity using both NDVI and vegetation volume. This buffer is larger than those used in other studies^32^, who found a 50 m buffer to be the most suitable. However, their surveys were based on a 50 m point count at each observation point^32^. Here, we used acoustic recordings with automated identification via BirdNET to determine the species occurring at our sites. The range at which an acoustic recorder can detect a species varies based on the environment, the traits of the species’ vocalisations^64^, and the recording hardware used^65^. Nonetheless, acoustic recorders typically detect species within a range of less than 100 m^66^, yet likely larger than 50 m, especially when combined with automated identification using BirdNET^20^. Therefore, at other buffer sizes, we may be seeing reduced explanatory power due to a mismatch in the scales of the biodiversity metrics and the vegetation measures, such as when vegetation data are only available for a 50 m buffer while acoustic recorders capture diversity within a 100 m radius. Conversely, irrelevant variability may be included if the buffer is too large, diminishing its explanatory power. This aligns with previous findings that local conditions are more important than the surrounding environment for bird diversity^42,43^, suggesting that while very small buffers may miss important resources, larger buffers incorporate less influential landscape features. It is worth noting that while we used the same buffer for all species, there is likely an effect of scale when investigating species individually, as others have found^34^. Thus, our findings suggest that using passive acoustic monitoring, a 100 m buffer effectively describes the spatial relationship between vegetation characteristics and overall bird diversity.

### NDVI as a better predictor of bird diversity

Although vegetation volume was expected to be a superior predictor of bird diversity, given its ability to describe urban vegetation in three dimensions, we found NDVI to be more effective. This is especially interesting considering NDVI and vegetation volume correlate well at the smaller buffers, including the 100 m buffer. While NDVI and vegetation volume show strong correlations at smaller buffer sizes (e.g., 100 m), this correlation weakens at larger scales. Irrespective of this divergence at larger scales, NDVI consistently explained more variance in our diversity metrics than vegetation volume, even at smaller buffer sizes where the two measures are highly correlated. This suggests that the additional information contained in NDVI values, beyond just vegetation presence and volume, contributes meaningfully to its predictive power for bird diversity.

Here, we suggest two potential explanations for this: (1) as suggested above, NDVI also quantifies properties of the non-vegetated pixels, and (2) NDVI quantifies additional properties of the vegetation beyond volume. Regarding explanation one, a key difference between our NDVI and vegetation volume maps is that NDVI provides values area-wide. In contrast, the vegetation volume map only provides data for pixels identified as having vegetation. Since NDVI provides a reflectance value for all surfaces, it may capture aspects of the urban environment that our vegetation volume measure does not. This distinction is important because non-vegetated areas in cities also provide resources for urban birds, such as nesting sites^67^ and potential food sources^68,69^. In addition, NDVI captures water areas, which are not included in the vegetation volume measure. Non-vegetated areas also include buildings, roads, and other structures that may act as barriers to movement^70^, areas of increased mortality^71^ and sources of disturbance that may affect species occurrence or activity at our sites. Regarding explanation two, NDVI can contain additional information about the vegetation that vegetation volume does not, for example, vegetation density or its physiological status^36,40^. If these vegetation properties are relevant for describing the habitat for birds, this could explain the higher predictive power of NDVI-based models. Obviously, the two explanations are not exclusive and can jointly contribute to the higher explanatory power of NDVI.

### Community composition

Our NMDS and environmental fit showed a clear shift in community composition along a gradient of urban green. The habitat preferences of the species driving our community composition also support this, with species more associated with human-modified landscapes opposite those more associated with forest or dense vegetation. This gradient, reflected primarily in NMDS1, demonstrates a continuum from open, urban habitats to densely vegetated areas. This underscores the strong influence of urban habitat structure on bird communities^15,72^. Consistent patterns were observed in an ordination using a presence-absence community matrix, supporting that shifts in community composition along vegetation gradients rather than sole differences in species’ vocal activity. While both vegetation volume and NDVI were good predictors for community composition, vegetation volume proved to be slightly better here. The alignment of both vectors towards the forest-associated species in the ordination space further emphasises the importance of vegetation occurrence and volume in shaping urban bird communities.

The better performance of vegetation volume in predicting community composition than diversity may indicate that the volume of vegetated habitat available is more important in determining which species can inhabit an area rather than how many. The relationship between vegetation volume and community composition may be explained by its representation of the occurrence of trees, which contribute to the species-specific habitat requirements (forest or dense vegetation^73^). These habitat requirements include factors such as preferred nesting heights, foraging strategies, and predator avoidance behaviours that are crucial for determining species presence^74^. The clustering of forest-associated species like the Eurasian treecreeper and Eurasian bullfinch in the high vegetation volume area supports this. The areas of high vegetation volume are found mainly in remnant forest areas and large parks, such as the English Garden. This is consistent with other studies findings on community composition in urban areas, showing an increase in the number of forest specialists in areas with remnant forests^72^.

### NDVI as a planning and conservation tool

We demonstrated that NDVI could reasonably predict bird diversity across an urban environment. It’s worth noting that our study represents a Western European city, and results may vary in other regions. However, the significance of our findings extends beyond this geographical constraint. Our study provides a robust methodological framework that can be adapted and tested in urban environments globally. Additionally, our findings contributed to a growing body of evidence supporting NDVI’s utility in predicting urban bird diversity^32–35^. The relationships between NDVI and biodiversity metrics are inherently complex. Previous studies reported varying strengths of correlation. While we observed similar patterns to previous studies, our results were, in some cases, slightly better. Notably, while Leveau (2019) did not find a link between abundance and NDVI, we identified a significant relationship between NDVI and Vocal Activity Rate, which may represent abundance for many species. In our models, partial R² values for mean NDVI ranged from 0.141 to 0.266, comparable to or exceeding those reported by Benedetti et al.^32^, whose marginal R^2^ ranged from 0.006 to 0.219. While these values may appear modest, they reflect the multifaceted nature of urban ecosystems, where bird diversity is influenced by numerous factors beyond vegetation alone.

Despite these limitations, NDVI remains valuable as an accessible and standardized predictor for rapid biodiversity assessment in urban planning contexts, particularly when more detailed ecological data are unavailable. Therefore, this approach offers a valuable tool for modelling potential changes in biodiversity due to urban conservation or building projects. Our method is compelling as NDVI measures are widely available for cities worldwide (e.g. WorldView-3 https://earth.esa.int/eogateway/missions/worldview-3), and as our work showed, accurate bird diversity models can be produced even with limited bird monitoring data. Such predictive maps can help identify areas of high bird diversity that should be protected and areas of low diversity that could benefit from improvement. Urban planners and conservationists can use this information to guide efforts, minimising impacts on biodiversity-rich areas and ensuring that current diversity is either maintained or improved^32^. By assigning average NDVI values to different urban features, planners can model how proposed changes might affect the overall NDVI map, allowing them to predict and quantify potential consequences for bird diversity. In addition, these maps can be used to identify and link areas of high diversity, creating wildlife corridors within the urban fabric^75^. Ultimately, this approach allows urban planners to put a numerical value on potential biodiversity losses or gains, providing a powerful tool for balancing urban development with ecological conservation.

However, using NDVI as a metric might be challenging as it does not provide something tangible that can be easily interpreted or implemented. For example, while an NDVI value of 0.6 may indicate high bird diversity, it does not provide the information a landscape architect needs to determine the exact number or area of trees or shrubs required to achieve this goal. This is important, as it has been shown that while urban green generally equates to more diversity, the composition of that green and other local features have a significant impact on the species found there^42^. Additionally, as our community composition shows, species react differently to increasing green, and for conservation efforts targeting a specific species, just increasing NDVI will likely not have the desired outcomes. Furthermore, accurately predicting a site’s NDVI and, therefore, bird diversity following certain interventions could prove challenging. Consequently, we suggest several avenues for future research to improve the applicability of this type of modelling in urban planning.

### Future research

While our predictive models for NDVI showed reasonable accuracy, they deviate from the actual values, possibly due to the complexity of urban ecosystems where a simple measure such as NDVI may not represent all factors influencing local diversity. Increasing the number of observation points will provide more training data and produce more robust models. Additionally, investigating what non-vegetative NDVI values represent in the urban environment could provide insights into why NDVI proved to be a better predictor than vegetation volume and help to advance a mechanistic understanding of why and where bird species can live in cities. The same holds true for additional vegetation properties that are (partially) represented by NDVI but not vegetation volume, like vegetation density, structure, or distribution. Further, understanding what NDVI values represent would allow concrete recommendations to be made for urban planners to implement. This could also include using indices explicitly developed for urban environments^76^. Furthermore, including additional measures and variables (e.g. building and neighbourhood typologies^77^, vegetation types^42^, water bodies^78^) could significantly improve the predictive power of the models, leading to more comprehensive and accurate assessments of urban biodiversity and a refined understanding of drivers of urban biodiversity. Lastly, as our community analysis suggested, investigating individual bird species or functional groups by building species-specific models could provide valuable insights into the drivers of urban bird diversity and inform targeted conservation efforts.

## Conclusion

We found compelling evidence for the effectiveness of NDVI as a predictor for urban bird diversity, outperforming vegetation volume in all diversity measures except community composition, further supporting previous research showing that NDVI is a good predictor for bird diversity. Contrary to our hypothesis, the better performance of NDVI may be due to its ability to capture a broader range of urban environmental factors, including vegetation density or physiological status and information on non-vegetated areas that provide resources or act as barriers for birds. While NDVI is proving to be a powerful tool for predicting bird diversity across an entire city, its practical application in urban planning and conservation faces challenges due to the abstract nature of NDVI values. Future research investigating species-specific differences and finer descriptors of the urban fabric may give more insight into the drivers of urban bird diversity and improve assessment tools. As urbanisation continues to impact biodiversity globally, refining our understanding and application of ecological models for urban planning will be crucial to developing more biodiverse cities.

## Electronic supplementary material

Below is the link to the electronic supplementary material.

Supplementary Material 1

## Data Availability

The research data supporting the main findings of this study are provided in the supplementary information files included with this manuscript.
