# Supplementary material for: NDVI and vegetation volume as predictors of urban bird diversity
Source: Sci Rep. 2025 Apr 15;15:12863. doi: 10.1038/s41598-025-96098-0 (PMC11997212; doi:10.1038/s41598-025-96098-0)
Supplement: Supplementary file 1 — Supplementary Material 1 [file 41598_2025_96098_MOESM1_ESM.pdf]

NDVI and vegetation volume as predictors of urban bird diversity: Supplement S1

Andrew J. Fairbairn, Sophia Katholnigg, Tobias Leichtle, Lisa Merkens, Louis Schroll, Wolfgang W. Weisser, Sebastian T. Meyer

Site summary statistics

Supplementary Table S1 Summary statistics of 86 sites acoustically monitored in Munich, Germany. Week refers to the specific week number of the year when recordings started; days is the total number of days recorded. For vegetation volume and NDVI, only the values in the 100m buffer are shown. Biodiversity metrics include species richness, vocal activity rate (VAR), and Shannon diversity index.

site	week	days	vegetation volume	NDVI	richness	VAR	Shannon
1	14	7	1.65	0.23	28	92.29	2.61
2	14	7	2.07	0.35	29	105.86	2.44
3	14	7	1.53	0.25	20	59.43	2.43
5	14	7	0.36	0.27	39	161	2.69
6	14	7	2.49	0.3	23	61.14	2.12
7	14	7	2.02	0.39	35	100.57	2.9
8	14	7	2.92	0.45	39	190.29	2.79
9	14	6.96	1.37	0.46	42	224.62	2.9
11	14	7	4.9	0.42	27	122.71	2.13
12	14	7	3.18	0.47	37	160.57	2.62
13	14	7	2.19	0.44	32	106.71	2.44
14	14	7	1.48	0.36	40	165.57	2.78
16	14	7	11.02	0.7	38	273	2.76
17	14	7	7.93	0.61	26	78.29	2.59
18	14	5	8.11	0.59	42	158	2.94
19	14	7	10.34	0.67	42	193.86	2.92
21	14	7	12.55	0.71	38	271.57	2.72
24	14	7	4.26	0.55	45	272.86	2.94
25	29	7	0.18	0.12	8	25.86	1.26

26	27	6	1.15	0.2	9	24	1.06
27	31	7	1.49	0.25	9	7.14	1.72
28	31	7	1.15	0.25	23	34	2.59
29	27	7	1.04	0.28	26	83.43	2.17
30	29	7	1.9	0.27	8	9.71	1.41
31	27	7	0.73	0.23	18	47	1.78
32	29	3.83	2.25	0.35	23	44.35	2.8
33	31	7	2.28	0.53	34	97	2.81
34	31	7	0.67	0.23	5	2	1.55
35	27	7	0.92	0.26	28	56	2.36
36	29	7	1.07	0.63	27	42.71	2.82
37	27	7	1.89	0.5	36	111.86	3.05
39	31	7	1.06	0.58	39	71.43	2.97
40	27	7	2.01	0.27	15	68.86	1.16
41	29	6	3.47	0.34	21	39	2.31
42	31	7	0.44	0.31	19	20.29	2.42
43	31	7	1.89	0.32	29	47.57	3
44	27	7	2.38	0.38	29	58.57	2.83
45	29	3.83	2.57	0.37	15	44.61	1.84
46	27	7	0.49	0.36	36	133.29	2.2
47	29	3.83	1.35	0.34	20	109.57	2.38
48	31	5	3.07	0.42	19	22.2	2.54
49	27	7	0.87	0.32	24	75.43	2.38
50	31	7	0.29	0.43	29	68	2.54
51	29	7	1.35	0.4	36	121.14	2.9
52	29	7	0.59	0.48	44	111.29	2.98
53	31	7	0.03	0.24	17	34.43	1.95
54	27	7	4.62	0.68	48	272.43	2.83
55	29	3.83	8.57	0.51	16	40.43	2.26
56	31	7	2.75	0.3	15	15.29	2.44
57	27	7	4.16	0.41	23	70.14	2.22
58	27	7	4.67	0.48	27	69.14	2.53

59	29	3.83	4.18	0.49	28	93.13	2.52
60	31	7	3.48	0.48	26	75.71	2.71
62	31	7	2.3	0.44	32	84	2.7
63	27	7	2.92	0.58	30	148	2.68
64	31	7	0.68	0.46	20	33.86	2.18
65	27	6.83	2.71	0.59	38	168.29	2.79
66	29	7	2.1	0.45	40	168.86	2.95
67	29	6.58	6.01	0.62	32	118.48	2.81
68	31	7	0.23	0.34	7	7.14	1.49
70	29	7	6.56	0.5	17	44.14	2.15
71	31	7	9.27	0.58	20	54.57	2.53
72	27	5	6.22	0.51	34	212.4	2.42
73	31	7	2.02	0.47	27	38.14	2.91
74	27	7	3.14	0.48	33	113.86	2.39
75	29	7	3.18	0.42	30	93.71	2.8
76	29	3.67	2.53	0.47	33	118.64	2.94
77	29	2.58	5.35	0.59	22	82.84	2.76
78	31	7	11.09	0.64	26	59.43	2.58
79	27	7	6.09	0.59	37	154.29	2.8
80	31	7	6.71	0.63	29	41.43	2.79
81	27	7	0.53	0.4	33	94.71	2.73
82	29	3.83	4.94	0.55	10	13.04	2.03
83	31	7	2.04	0.63	35	73.29	2.79
86	29	3.83	12.88	0.7	20	78.26	2.55
87	31	7	8.25	0.45	9	5	2
88	27	7	2.93	0.32	16	33.86	1.21
89	27	7	7.64	0.67	35	201.86	2.67
91	31	7	5.89	0.56	31	37.57	2.95
92	31	6	2.73	0.46	23	48.67	2.11
94	29	3.83	9.95	0.62	27	119.22	2.49
95	31	7	10.75	0.7	23	60	2.4
96	27	7	8.85	0.6	35	128.57	2.9

97	31	7	0.93	0.47	18	27.57	2.07
98	27	7	6.81	0.69	33	134.43	2.58
99	29	6.54	8.99	0.66	21	68.03	2.29

Correlation of mean EVIN and NDVI and mean NDVI and log mean vegetation volume.

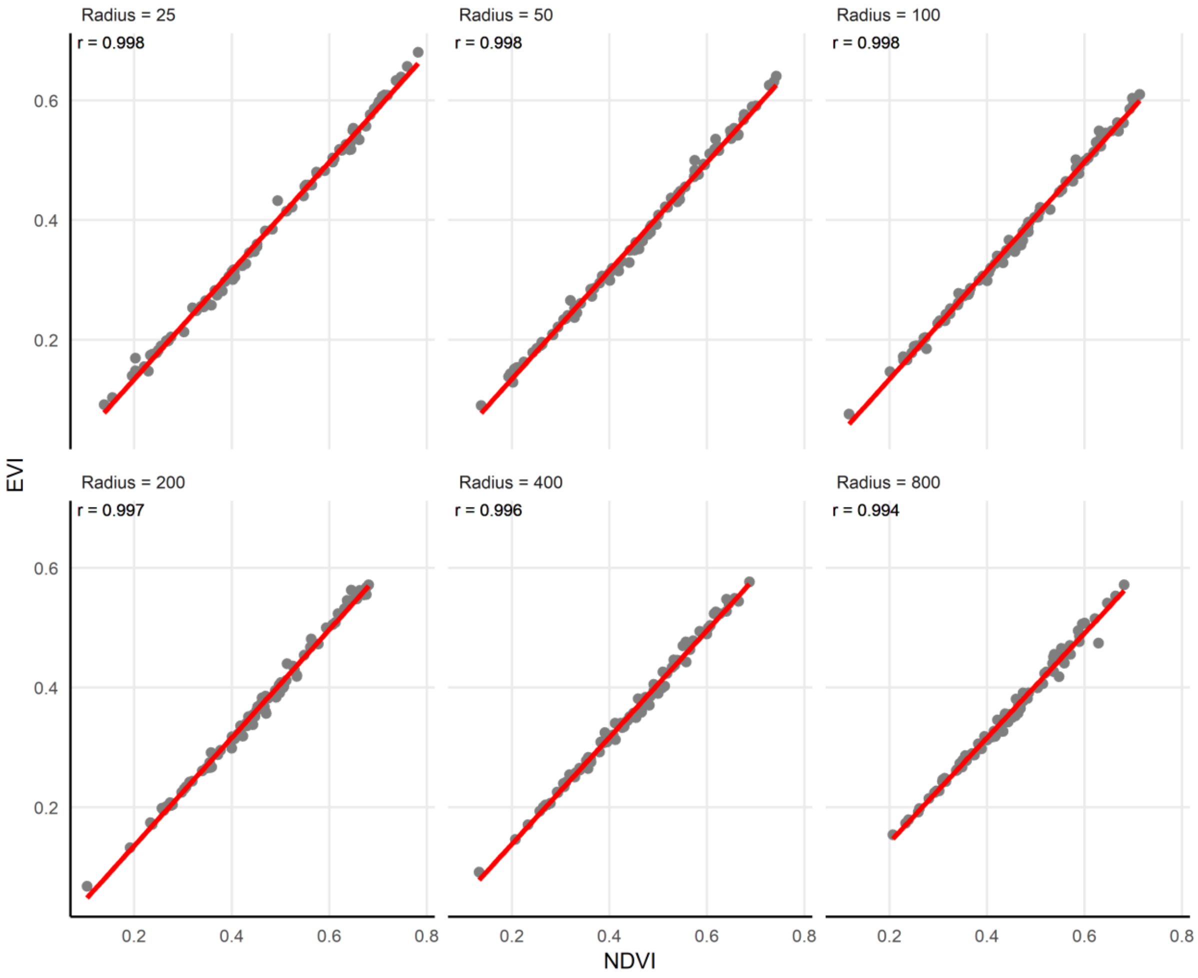

Supplementary Fig. S1 Correlation between Normalized Difference Vegetation Index (NDVI) and Enhanced Vegetation Index (EVI) at six different spatial scales (radii: 25m, 50m, 100m, 200m, 400m, and 800m). Red lines indicate linear regression fits. Pearson's correlation coefficient (r) is displayed in the top left corner of each subplot.

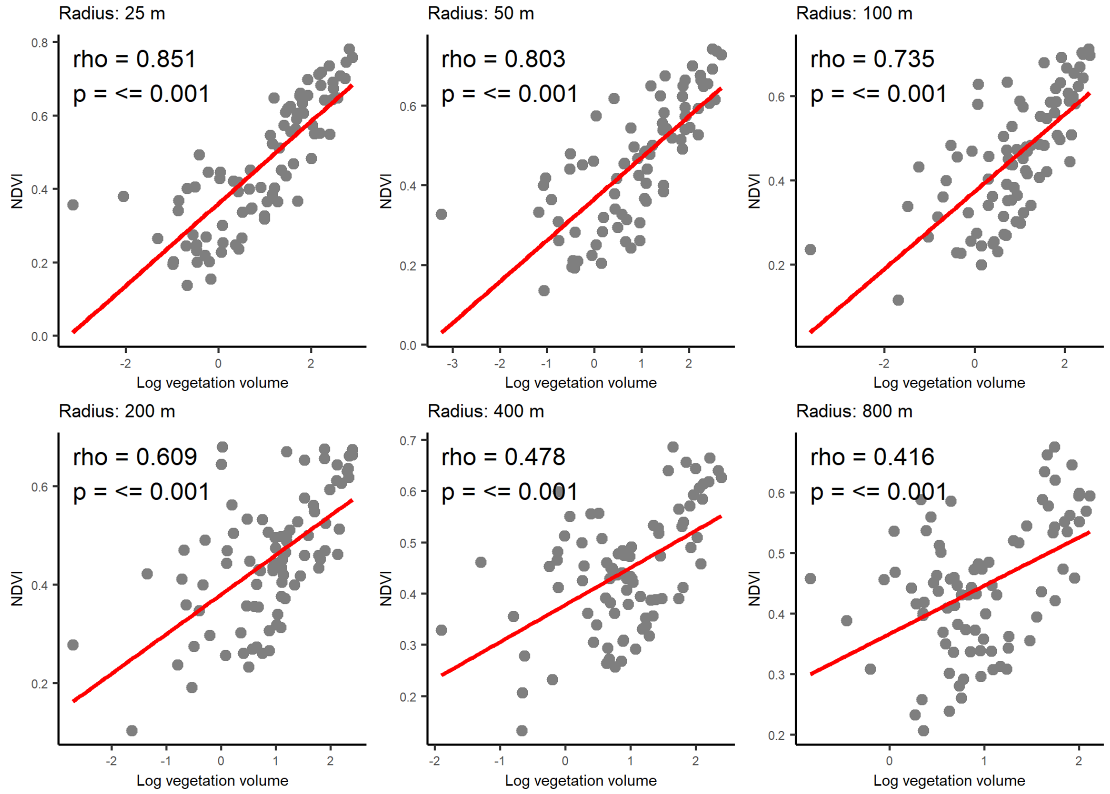

Supplementary Fig. S2 Relationship between log-transformed vegetation volume and Normalized Difference Vegetation Index (NDVI) at six different spatial scales (radii: 25m, 50m, 100m, 200m, 400m, and 800m). Red lines indicate linear regression fits. Spearman's rank correlation coefficients (rho) and corresponding p-values are displayed in the top-left corner of each subplot

Species detected in Munich, Germany from 86 sites

Supplementary Table S2 Species found via acoustic monitoring and automatic identification with BirdNET on 86 sites in Munich, Germany. N sites is the number of sites the species was detected on. Min, mean, sd, and max vocal activity rate (VAR) are presented. Dominant habitat is also presented.

		VAR				
scientific name	n sites	min	mean ± sd	max	total	habitat
Accipiter nisus	3	0.60	2.34 ± 1.51	3.29	7.02	Forest
Actitis hypoleucos	22	0.14	0.44 ± 0.47	2.43	9.68	Wetland
Aegithalos caudatus	27	0.14	0.9 ± 1.16	5.43	24.4	Forest
Alauda arvensis	3	3.86	5.94 ± 1.9	7.57	17.8	Grassland
Alcedo atthis	5	0.14	0.43 ± 0.23	0.71	2.16	Riverine
Anas crecca	10	0.29	0.37 ± 0.12	0.57	3.69	Wetland
Anas platyrhynchos	9	0.29	1.06 ± 0.82	2.86	9.55	Wetland
Anser anser	15	0.14	1.52 ± 2.03	7.71	22.8	Wetland
Anthus trivialis	54	0.14	1.04 ± 1.58	11.14	56.3	Woodland
Apus apus	59	0.14	7.04 ± 10.68	51.57	415	Human Modified
Ardea cinerea	40	0.14	0.61 ± 0.38	1.57	24.5	Wetland
Branta canadensis	1	0.29	0.29 ± NA	0.29	0.29	Grassland

Buteo buteo	24	0.29	0.92 ± 0.91	4.29	22.1	Grassland
Carduelis carduelis	71	0.26	6.83 ± 9.52	44.14	485	Woodland
Certhia brachydactyla	68	0.14	5.5 ± 6.82	38.14	374	Forest
Certhia familiaris	6	0.43	0.94 ± 0.69	2.14	5.66	Forest
Charadrius dubius	4	0.14	0.35 ± 0.24	0.57	1.41	Wetland
Chloris chloris	65	0.14	7.19 ± 9.27	43.71	468	Woodland
Ciconia ciconia	3	0.14	0.52 ± 0.36	0.86	1.57	Grassland
Coccothraustes coccothraustes	66	0.29	2.12 ± 2.16	9.13	140	Forest
Columba livia	37	0.17	0.85 ± 1.33	7.29	31.5	Human Modified
Columba oenas	11	0.29	1.05 ± 0.98	3.29	11.5	Woodland
Columba palumbus	64	0.14	3.06 ± 4	24.30	196	Woodland
Corvus corax	7	0.14	0.25 ± 0.06	0.29	1.77	Forest
Corvus cornix	53	0.29	4.98 ± 5.29	32.00	264	Human Modified
Corvus corone	78	0.29	2.87 ± 2.38	11.71	224	Human Modified
Corvus frugilegus	65	0.29	2.89 ± 4.69	28.71	188	Grassland
Corvus monedula	4	0.14	1.29 ± 1.91	4.14	5.14	Human Modified
Curruca curruca	3	0.14	0.24 ± 0.08	0.29	0.71	Shrubland
Cyanistes caeruleus	66	0.14	4.67 ± 7.2	38.86	308	Forest
Delichon urbicum	23	0.14	1.78 ± 2.8	11.86	40.9	Human Modified
Dendrocopos major	74	0.29	6.61 ± 7.13	31.00	489	Woodland
Dendrocoptes medius	6	0.29	0.45 ± 0.28	1.00	2.72	Woodland
Dryobates minor	9	0.14	0.37 ± 0.19	0.71	3.29	Forest
Dryocopus martius	10	0.14	0.56 ± 0.49	1.57	5.58	Forest
Emberiza citrinella	8	0.29	13.43 ± 22	56.60	107	Shrubland
Erithacus rubecula	61	0.29	4.45 ± 4.71	22.80	272	Forest
Falco tinnunculus	30	0.14	1.56 ± 1.72	7.14	46.9	Shrubland
Ficedula hypoleuca	22	0.29	0.68 ± 0.49	2.14	15	Forest
Fringilla coelebs	39	0.14	5.52 ± 10.19	47.57	215	Forest
Fulica atra	29	0.29	1.41 ± 3.79	21.00	41	Wetland
Gallinula chloropus	25	0.14	0.64 ± 0.5	2.01	15.9	Wetland
Garrulus glandarius	39	0.14	1.02 ± 0.95	4.00	39.6	Forest
Hirundo rustica	10	0.20	1.03 ± 0.88	2.57	10.3	Human Modified
Larus michahellis	8	0.14	0.46 ± 0.29	1.04	3.71	Human Modified
Lophophanes cristatus	15	0.17	0.72 ± 1	3.86	10.8	Woodland
Motacilla alba	15	0.14	2.23 ± 2.94	11.29	33.4	Human Modified
Motacilla cinerea	27	0.14	0.64 ± 0.5	2.09	17.2	Riverine
Motacilla flava	3	0.29	1 ± 0.71	1.71	3	Grassland
Muscicapa striata	35	0.14	1.46 ± 1.33	6.43	50.9	Forest
Nycticorax nycticorax	15	0.29	0.94 ± 0.8	3.00	14.1	Wetland
Oriolus oriolus	3	0.57	0.62 ± 0.08	0.71	1.86	Woodland

Parus major	64	0.14	7.09 ± 10.84	36.43	454	Woodland
Passer domesticus	11	0.14	1.74 ± 1.76	5.71	19.1	Human Modified
Passer montanus	12	0.17	3.46 ± 5.01	16.71	41.5	Woodland
Periparus ater	13	0.26	4.92 ± 7.28	21.86	64	Forest
Phasianus colchicus	20	0.29	1.43 ± 3.04	13.94	28.6	Human Modified
Phoenicurus ochruros	36	0.14	2.8 ± 3.89	18.86	101	Human Modified
Phoenicurus phoenicurus	17	0.14	0.97 ± 1.19	4.14	16.5	Woodland
Phylloscopus collybita	59	0.17	8.78 ± 11.76	46.00	518	Forest
Pica pica	46	0.14	3.35 ± 4.03	14.73	154	Human Modified
Picus viridis	78	0.14	4.11 ± 4.04	20.86	321	Forest
Poecile palustris	46	0.14	1.62 ± 1.67	6.57	74.4	Forest
Prunella modularis	3	0.57	1.1 ± 0.58	1.71	3.31	Forest
Pyrrhula pyrrhula	12	0.29	1.52 ± 2.13	7.29	18.3	Forest
Rallus aquaticus	6	0.20	0.69 ± 0.93	2.57	4.15	Wetland
Regulus ignicapilla	18	0.29	0.96 ± 1.1	4.71	17.3	Forest
Regulus regulus	6	0.29	2.64 ± 3.48	9.43	15.8	Forest
Serinus serinus	9	0.14	1.59 ± 3.91	12.00	14.3	Forest
Sitta europaea	47	0.14	5.41 ± 9.91	47.00	254	Forest
Spinus spinus	15	0.29	0.52 ± 0.55	2.43	7.86	Forest
Streptopelia decaocto	29	0.29	2.09 ± 3.79	18.71	60.7	Human Modified
Strix aluco	39	0.43	4.44 ± 3.2	13.00	173	Forest
Sturnus vulgaris	2	0.29	0.43 ± 0.2	0.57	0.86	Human Modified
Sylvia atricapilla	46	0.14	5.47 ± 10.3	44.20	252	Woodland
Sylvia borin	3	0.40	0.66 ± 0.42	1.14	1.98	Woodland
Tachybaptus ruficollis	1	2.86	2.86 ± NA	2.86	2.86	Wetland
Tringa nebularia	3	0.14	0.37 ± 0.21	0.57	1.1	Wetland
Tringa ochropus	33	0.14	0.53 ± 0.42	2.14	17.3	Wetland
Troglodytes troglodytes	20	0.14	3.6 ± 3.57	13.29	72	Forest
Turdus merula	64	0.14	4.81 ± 6.92	26.14	308	Forest
Turdus philomelos	62	0.29	0.87 ± 0.79	5.14	54.1	Forest
Turdus pilaris	32	0.14	1.11 ± 1.64	8.14	35.4	Forest
Turdus viscivorus	12	0.29	2.56 ± 7.56	26.57	30.7	Forest
Upupa epops	9	0.29	0.49 ± 0.2	0.86	4.43	Grassland
Vanellus vanellus	5	0.40	1.88 ± 2.35	6.00	9.4	Wetland

ANOVA results from linear models for radius selection analyses

Supplementary Table S3 ANOVA results from linear models for three diversity metrics (vocal activity rate (VAR), species richness, and Shannon diversity) and log mean vegetation volume and mean NDVI at six distance radii (25m, 50m, 100m, 200m, 400m, 800m) from 86 observation points in Munich, Germany.

radius	diversity metric	vegetation volume	covariates	adjusted R2
25	Richness	$F_{1,82}$=7.34; p=0.008	week $F_{1,82}$=26.59; p≤0.001<br>distance $F_{1,82}$=23.59; p≤0.001	0.39
	VAR	$F_{1,82}$=14.1; p≤0.001	week $F_{1,82}$=51.44; p≤0.001<br>distance $F_{1,82}$=8.18; p=0.005	0.45
	Shannon	$F_{1,82}$=10.58; p=0.002	week $F_{1,82}$=4.18; p=0.044<br>distance $F_{1,82}$=13.09; p≤0.001	0.23
50	Richness	$F_{1,82}$=7.98; p=0.006	week $F_{1,82}$=26.78; p≤0.001<br>distance $F_{1,82}$=23.76; p≤0.001	0.40
	VAR	$F_{1,82}$=14.64; p≤0.001	week $F_{1,82}$=51.73; p≤0.001<br>distance $F_{1,82}$=8.22; p=0.005	0.46
	Shannon	$F_{1,82}$=15.22; p≤0.001	week $F_{1,82}$=4.39; p=0.039<br>distance $F_{1,82}$=13.75; p≤0.001	0.26
100	Richness	$F_{1,82}$=8.90; p=0.004	week $F_{1,82}$=27.05; p≤0.001<br>distance $F_{1,82}$=24; p≤0.001	0.40
	VAR	$F_{1,82}$=13.02; p≤0.001	week $F_{1,82}$=50.86; p≤0.001<br>distance $F_{1,82}$=8.09; p=0.006	0.45
	Shannon	$F_{1,82}$=16.28; p≤0.001	week $F_{1,82}$= 4.43; p=0.038<br>distance $F_{1,82}$=13.9; p≤0.001	0.27
200	Richness	$F_{1,82}$=5.39; p=0.02	week $F_{1,82}$=26.01; p≤0.001<br>distance $F_{1,82}$=23.08; p≤0.001	0.38
	VAR	$F_{1,82}$=8.4; p=0.005	week $F_{1,82}$=48.39; p≤0.001<br>distance $F_{1,82}$=7.69; p=0.007	0.42
	Shannon	$F_{1,82}$=9.36; p=0.003	week $F_{1,82}$=4.12; p=0.046<br>distance $F_{1,82}$=12.92; p=0.003	0.22
400	Richness	$F_{1,82}$=5.6; p=0.02	week $F_{1,82}$=26.07; p≤0.001<br>distance $F_{1,82}$=23.13; p≤0.001	0.38
	VAR	$F_{1,82}$=9.14; p=0.003	week $F_{1,82}$=48.78; p≤0.001<br>distance $F_{1,82}$=7.76; p=0.007	0.42
	Shannon	$F_{1,82}$=6.1; p=0.016	week $F_{1,82}$=3.97; p=0.05<br>distance $F_{1,82}$=12.46; p≤0.001	0.19
800	Richness	$F_{1,82}$=5.72; p=0.02	week $F_{1,82}$=26.17; p≤0.001<br>distance $F_{1,82}$=23.16; p≤0.001	0.38
	VAR	$F_{1,82}$=7.53; p=0.007	week $F_{1,82}$=47.92; p≤0.001<br>distance $F_{1,82}$=7.62; p=0.007	0.41
	Shannon	$F_{1,82}$=4.01; p=0.048	week $F_{1,82}$=3.88; p=0.052<br>distance $F_{1,82}$=12.17; p≤0.001	0.17

radius	diversity metric	NDVI	covariates	adjusted R2

25	Richness	$F_{1,82}$=8.31; p=0.005	week $F_{1,82}$=26.87; p≤0.001<br>distance $F_{1,82}$=23.85; p≤0.001	0.40
	VAR	$F_{1,82}$=17.06; p≤0.001	week $F_{1,82}$=53.02; p≤0.001<br>distance $F_{1,82}$=8.43; p=0.005	0.47
	Shannon*	$F_{1,82}$=19.26; p≤0.001	week $F_{1,82}$=4.57; p=0.036<br>distance $F_{1,82}$=14.32; p≤0.001	0.29
50	Richness	$F_{1,82}$=15.35; p≤0.001	week $F_{1,82}$=28.97; p≤0.001<br>distance $F_{1,82}$=25.71; p≤0.001	0.44
	VAR	$F_{1,82}$=24.69; p≤0.001	week $F_{1,82}$=57.11; p≤0.001<br>distance $F_{1,82}$=9.08; p=0.003	0.51
	Shannon*	$F_{1,82}$=32.79; p≤0.001	week $F_{1,82}$=5.18; p=0.025<br>distance $F_{1,82}$=16.24; p≤0.001	0.38
100	Richness	$F_{1,82}$=23.16; p≤0.001	week $F_{1,82}$=31.29; p≤0.001<br>distance $F_{1,82}$=27.77; p≤0.001	0.48
	VAR	$F_{1,82}$=30.8; p≤0.001	week $F_{1,82}$=60.38; p≤0.001<br>distance $F_{1,82}$=9.6; p=0.003	0.53
	Shannon*	$F_{1,82}$=37.89; p≤0.001	week $F_{1,82}$=5.41; p=0.023<br>distance $F_{1,82}$=16.96; p≤0.001	0.40
200	Richness	$F_{1,82}$=19.77; p≤0.001	week $F_{1,82}$=30.29; p≤0.001<br>distance $F_{1,82}$=26.87; p≤0.001	0.47
	VAR	$F_{1,82}$=20.05; p≤0.001	week $F_{1,82}$=54.62; p≤0.001<br>distance $F_{1,82}$=8.69; p=0.004	0.49
	Shannon*	$F_{1,82}$=24.91; p≤0.001	week $F_{1,82}$=4.82; p≤0.031<br>distance $F_{1,82}$=15.12; p≤0.001	0.33
400	Richness	$F_{1,82}$=14.87; p≤0.001	week $F_{1,82}$=28.8; p≤0.001<br>distance $F_{1,82}$=25.56; p≤0.001	0.44
	VAR	$F_{1,82}$=9.75; p=0.002	week $F_{1,82}$=49.11; p≤0.001<br>distance $F_{1,82}$=7.81; p=0.006	0.43
	Shannon*	$F_{1,82}$=15.08; p≤0.001	week $F_{1,82}$=4.38; p=0.039<br>distance $F_{1,82}$=13.73; p≤0.001	0.26
800	Richness	$F_{1,82}$=13.51; p≤0.001	week $F_{1,82}$=28.42; p≤0.001<br>distance $F_{1,82}$=13.19; p≤0.001	0.43
	VAR	$F_{1,82}$=5.43; p=0.022	week $F_{1,82}$=46.8; p≤0.001<br>distance $F_{1,82}$=7.44; p=0.008	0.40
	Shannon*	$F_{1,82}$=11.74; p=0.001	week $F_{1,82}$=4.23; p=0.043<br>distance $F_{1,82}$=13.26; p≤0.001	0.24

* NDVI log-transformed

Community analysis

Supplementary information S1:

While we expected some differences between the presence-absence data (Jaccard distance) and the vocal activity rate (VAR, representing abundance via Bray-Curtis distance), visual inspection of the patterns revealed similarities between the two. The Mantel test confirmed a strong, statistically significant correlation between the two distance matrices (r = 0.8762, p = 0.001). Additionally, the Procrustes analysis showed a good overall fit between the two ordinations, with a root mean squared error (RMSE) of 0.2428, suggesting that the spatial patterns captured by both methods are closely aligned.

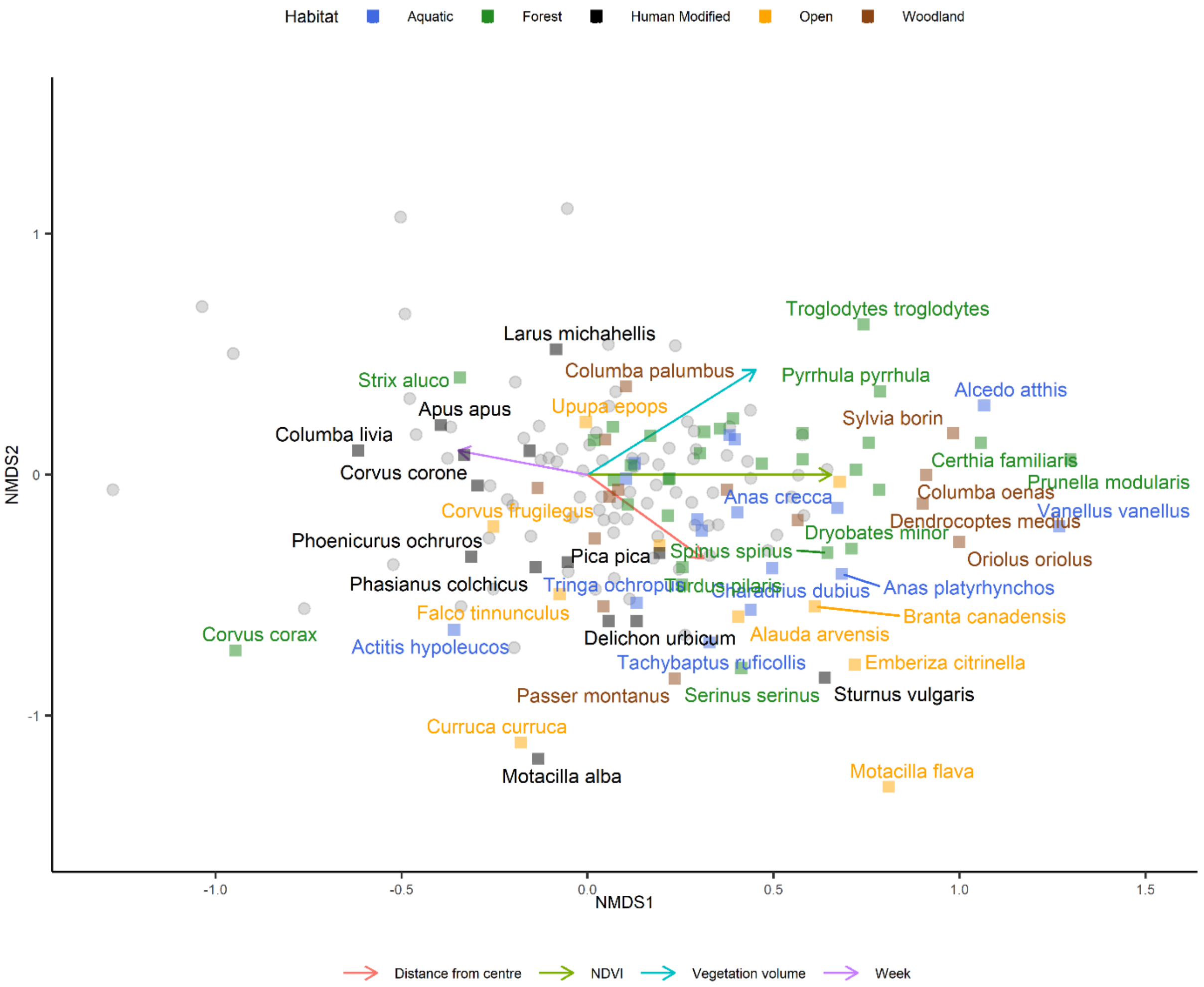

Supplementary Fig. S2 Non-metric multidimensional scaling (NMDS) ordination plot with environmental variables indicating the dissimilarity of communities across green mean NDVI and blue log total vegetation volume for 86 sites in Munich, Germany. Utilising presence-absence data and a Jaccard distance matrix. Grey points represent sites. Species points and labels are coloured by their dominant habitat type. Not all sites or species names are displayed.

Predictive modelling

Supplementary Table S4: ANOVA results from linear models for predicting three diversity metrics based on NDVI and the covariates of recording week and distance to the city centre.

model	variable	F and p value	adjusted r-squared
richness	week	$F_{1,65}$=22.81; p≤0.001	0.49
	distance	$F_{1,65}$=21.70; p≤0.001	
	NDVI	$F_{1,65}$=23.74; p≤0.001	
VAR	week	$F_{1,65}$=48.53; p≤0.001	0.54
	distance	$F_{1,65}$=5.14; p=0.003	
	NDVI	$F_{1,65}$=29.23; p≤0.001	
Shannon	week	$F_{1,65}$=4.17; p=0.045	0.42
	distance	$F_{1,65}$=13.88; p≤0.001	
	NDVI	$F_{1,65}$=33.38; p≤0.001	

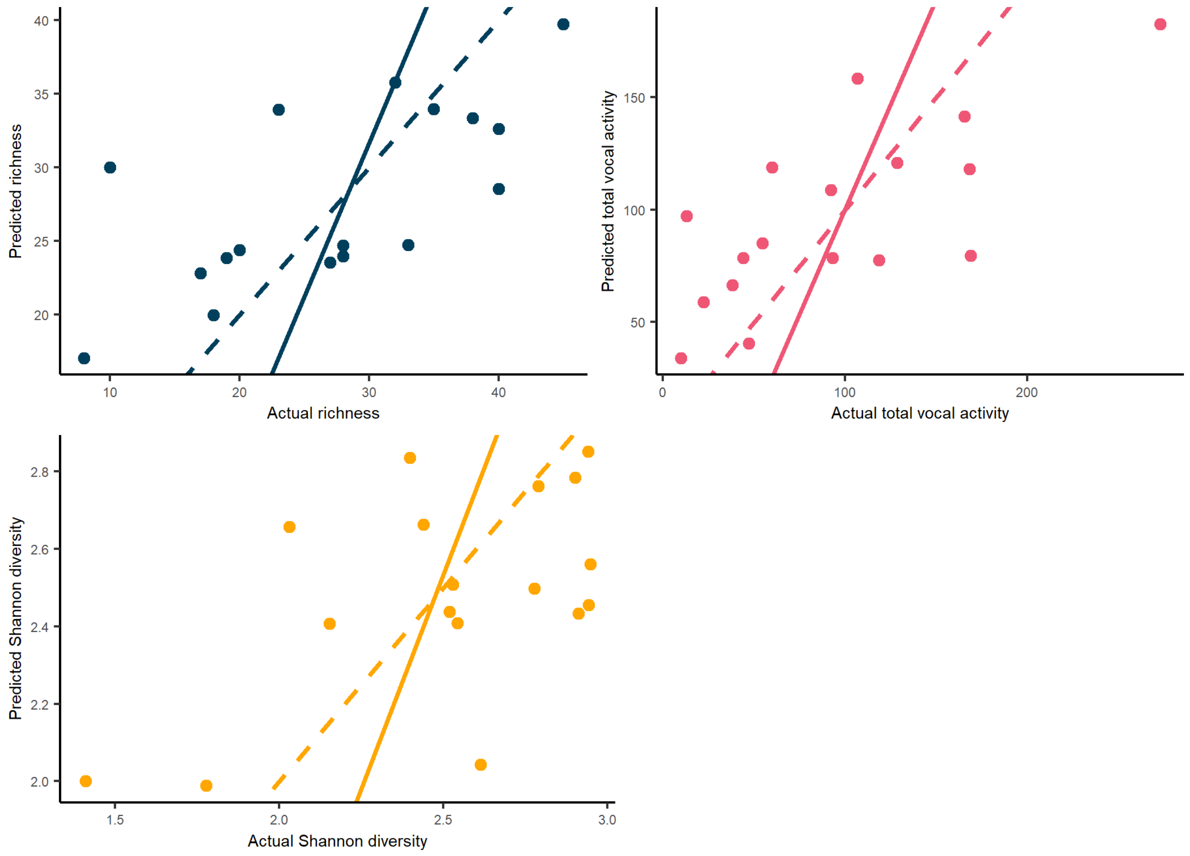

Supplementary Fig. S3 Predicted versus actual plots for models predicting four bird diversity metrics across the city of Munich, Germany. The solid lines represent the Standardized (reduced) major axis model II linear regression, and the dashed lines represent the perfect correlation of 45 degrees with an intercept of 0 and slope of 1

Supplementary Table S5: Reduced major axis regression for actual vs predicted results of predictive models for 17 predictions. R is the Pearson correlation coefficient. Two and one-tailed parametric p-values.

**Variable**	**r**	**r^2**	**2-tailed p-value**	**1-tailed p-value**
richness	0.68	0.46	0.002	0.001
VAR	0.73	0.53	0.001	0.000
Shannon diversity	0.57	0.32	0.018	0.009
